# Laparoscopic or open abdominal surgery with thoracotomy for patients with oesophageal cancer: ROMIO randomized clinical trial

**DOI:** 10.1093/bjs/znae023

**Published:** 2024-03-25

**Authors:** The ROMIO Study Group, Khurshid Akhtar, Bilal Alkhaffaf, Arun Ariyarathenam, Kerry Avery, Paul Barham, Adrian Bateman, Chloe Beard, Richard Berrisford, Jane M Blazeby, Natalie Blencowe, Alex Boddy, David Bowrey, Tim Bracey, Rachel C Brierley, Kate Briton, James Byrne, James Catton, Ram Chaparala, Sarah K Clark, Tonia Clarke, Jill Cooke, Graeme Couper, Lucy Culliford, Heidi Dawson, Chris Deans, Jenny L Donovan, Charlotte Ekblad, Jackie Elliott, David Exon, Stephen Falk, Naheed Farooq, Kirsty Garfield, Daisy M Gaunt, Fran Gill, Robert Goldin, Athanasia Gravani, George Hanna, Stephen Hayes, Rachael Heys, Carolyn Hindmarsh, Sandra Hollinghurst, William Hollingworth, Andrew Hollowood, Rebecca Houlihan, Benjamin Howes, Lucy Howie, Lee Humphreys, David Hutton, Rosina Jarvis, Marcus Jepson, Rebecca Kandiyali, Surinder Kaur, Philip Kaye, Jamie Kelly, Anni King, Jana Kirwin, Richard Krysztopik, Peter Lamb, Alistair Lang, Vivienne Lee, Sally Maitland, Nicholas Mapstone, Georgia Melia, Chris Metcalfe, Rachel Melhado, Aida Moure-Fernandez, Beena Nair, Joanna Nicklin, Fergus Noble, Sian M Noble, Abby O’Connell, Stephen Palmer, Simon Parsons, Kish Pursnani, Nicola Rea, Fiona Reed, Caoimhe Rice, Cathy Richards, Chris Rogers, Grant Sanders, Vicki Save, Chas Shaw, Michael Schiller, Rachel Schranz, Vinutha Shetty, Beverly Shirkey, Jo Singleton, Richard Skipworth, Joanne Smith, Christopher Streets, Dan Titcomb, Paul Turner, Sukhbir Ubhi, Tim Underwood, Cellins Vinod, Ravinder Vohra, Elizabeth M Ward, Rhian Warman, Neil Welch, Tim Wheatley, Katie White, Robin A Wickens, Paul Wilkerson, Alexandra Williams, Rob Williams, Natasha Wilmshurst, Newton A C S Wong

## Abstract

**Objective:**

This study investigated if hybrid oesophagectomy with minimally invasive gastric mobilization and thoracotomy enabled faster recovery than open surgery.

**Methods:**

In eight UK centres, this pragmatic RCT recruited patients for oesophagectomy to treat localized cancer. Participants were randomly allocated to hybrid or open surgery, stratified by centre and receipt of neoadjuvant treatment. Large dressings aimed to mask patients to their allocation for six days post-surgery. The authors present the intention-to-treat analysis of outcome measures from the first 3 months post-randomization, including the primary outcome, the patient-reported physical function scale of the EORTC QLQ-C30, and cost-effectiveness. Current Controlled Trials registration: ISRCTN 59036820 (feasibility study), 10386621 (definitive study).

**Findings:**

There was no evidence of a difference between hybrid (*n* = 267) and open (*n* = 266) surgery in average physical function over 3 months post-randomization: difference in means 2.1, 95% c.i. −2.0 to 6.2, *P* = 0.3. Complication rates were similar; for example, 88 (34%) participants in the open and 82 (32%) participants in the hybrid surgery groups experienced a pulmonary infection within 30 days. There was no evidence that hybrid surgery was more cost-effective than open surgery at 3 months.

**Conclusions:**

Patient-reported physical function in the 3 months post-randomization provided no evidence of a difference in recovery time between hybrid and open surgery, or a difference in cost-effectiveness. Both approaches to surgery were completed safely, with a similar risk of key complications, suggesting that surgeons who have a preference for one of the two approaches need not change their practice.

## Teaser

This pragmatic randomized controlled trial conducted in eight UK centres allocated patients with localized oesophageal cancer to hybrid oesophagectomy with minimally invasive gastric mobilization and thoracotomy, or open surgery. The primary outcome was patient-reported recovery in the 3 months following allocation, measured using the EORTC QLQ-C30 physical function subscale. We found no evidence of a difference in recovery time between hybrid and open surgery, and a similar risk of key complications.

## Introduction

Oesophageal cancer is the tenth most common cancer globally and causes one in 18 cancer deaths^1^. Oesophagectomy, with or without neoadjuvant therapy, is recommended for patients whose disease is confined to the oesophagus and the local lymph nodes, and who are fit to undergo surgery. The most common approach in the UK is the two-phase Ivor Lewis oesophagectomy, which involves incisions in both the abdomen and chest.

Minimally invasive approaches aim to reduce damage to healthy tissues and allow more rapid recovery, while maintaining the clinical benefits of open surgery. For oesophagectomy, two minimally invasive approaches are commonly used: hybrid or laparoscopically assisted oesophagectomy, where the chest phase uses open surgery and the abdominal phase uses laparoscopically assisted surgery; and totally minimally invasive oesophagectomy for both the abdominal and chest phases.

The use of minimally invasive techniques has been steady in England and Wales at around 50% of curative procedures since 2015. National Audit data from England and Wales, for patients diagnosed between April 2019 and March 2021, indicated that 33% of Ivor Lewis oesophagectomies were started as laparoscopically assisted, and 18% were started as totally minimally invasive^2,3^. National data for France between 2017 and 2019 showed greater adoption of minimally invasive approaches to Ivor Lewis oesophagectomy, with 56% of procedures being hybrid and 7% being totally minimally invasive^4^.

The impact of oesophagectomy on health-related quality of life is substantial and can persist for several years post-surgery^5^. If minimally invasive techniques can be demonstrated to lessen the impact of surgery, with significant benefit to patients, it will be important to explore their use in a greater proportion of cases. To date, two multicentre RCTs have shown that minimally invasive surgery may reduce postoperative complications, but had sample sizes too limited to confirm that minimally invasive methods achieve the same survival benefit^6,7^. The latter cannot be assumed when applying minimally invasive approaches to complex cancer surgery in the absence of evidence from RCTs^8^. The ROMIO study addresses this evidence gap, aiming to compare speed of recovery following hybrid and open oesophagectomy in a pragmatic RCT of more than 500 participants, with participants followed-up for at least 2 years for vital status, and clinical and patient-reported outcome measures. Here, outcomes up to 3 months post-randomization including the primary outcome, patient-reported postoperative recovery, are reported.

## Methods

### Study design and participants

The ROMIO study design has been described in detail elsewhere^9^. In brief, ROMIO is a pragmatic parallel group RCT comparing hybrid *versus* open oesophagectomy in patients with oesophageal cancer at eight National Health Service (NHS) hospitals in the UK. ROMIO began as an external pilot study at two centres (registered with Current Controlled Trials on 25 February 2013: ISRCTN 59036820) with recruitment commencing on 8 April 2013. Due to the success of the pilot, this initial phase was then adapted to an internal pilot study with recruitment continuing until funding was secured for the definitive trial^10^. Patients from the pilot phase were therefore included in the main trial analysis, with the exception of pilot-phase participants with high-grade dysplasia who, following changes to UK treatment recommendations during the pilot study period, did not meet the eligibility criteria for the main trial. The definitive trial was registered separately (31 May 2016, ISRCTN 10386621) and recruitment expanded to all eight centres from October 2016 until 21 August 2019, follow-up being completed on 31 August 2021.

Two ROMIO centres randomly allocated participants between three groups, the third group being part of a nested randomized IDEAL phase 2b substudy^11^, which will be reported separately.

Eligible adult patients had adenocarcinoma or squamous cell cancer localized in the oesophagus or oesophagogastric junction situated 5 cm below the cricopharyngeus and involving <4 cm of the stomach wall, and were referred for oesophagectomy (two- or three-phase) by the multidisciplinary cancer care team after any neoadjuvant therapy^9^. The decision for a two- or three-phase approach was at the discretion of the operating surgeon. Patients were invited to give written informed consent to participate in the study by surgeons who received training from an integrated QuinteT Recruitment Intervention^12^.

The South-West Frenchay Research Ethics Committee (main study reference 184167, pilot study reference 12/SW/0161) approved the study, with all versions of the protocol available from https://fundingawards.nihr.ac.uk/award/14/140/78. All participants provided written informed consent before taking part.

### Randomization and masking

Patients were randomly allocated in a 1:1 ratio according to a blocked (varying block size of six or eight) and stratified sequence. The two centres participating in the IDEAL phase 2b substudy randomly allocated three ways in a 1:1:1 ratio, with block sizes of six and nine. Stratification was by study centre and whether the patient underwent neoadjuvant treatment prior to surgery. The allocation sequence was computer-generated by a bespoke software program during the internal pilot, and post-pilot was generated by the study statistician who was not otherwise involved with participant recruitment, using Stata statistical software version 14.1 (StataCorp 2015, College Station, Texas, USA).

Patients who gave written informed consent to participate in the ROMIO study were logged into the study and only then was the patient randomized and the surgical team informed of the allocation through the database, so ensuring concealment of allocation. Participating surgeons performed both open and hybrid surgery, according to the allocation. Participants and hospital staff (not on the surgical team) were masked to allocation until after the assessment of pain 6 days post-surgery, by covering all the wound sites for both surgical approaches with the same-sized dressings.

### Procedures

The protocol for surgical quality assurance has been published and a detailed analysis of fidelity to allocated intervention will be reported separately^13^. Prior to participating in ROMIO, surgeons provided two anonymized unedited videos of laparoscopic cases, which had to meet the standard presented in a video assessment tool developed during the feasibility phase^13^.

In both study groups, two-phase (abdomen and chest) Ivor Lewis operations were expected, but with three-phases (abdomen, chest and neck) permitted. This may have been decided preoperatively or intraoperatively and this was flexible. Antibiotics and deep vein thrombosis prophylaxis were administered according to local hospital policies. Co-interventions such as perioperative analgesia were permitted according to the preferences of each centre.

For open surgery, transhiatal and thoracoabdominal approaches were prohibited. The location and length of incisions were at each surgeon’s discretion, and were recorded.

For the hybrid procedure, access to the abdominal cavity was achieved with several 12- or 5-mm incisions (as many as needed) and surgery performed laparoscopically. Laparoscopic transhiatal approaches were prohibited. Methods to create the pneumoperitoneum were at the surgeon’s discretion. If a feeding jejunostomy was placed, this could be performed laparoscopically or by creating an additional abdominal incision (maximum length of 8 cm).

For both open and hybrid surgery, complete gastric mobilization was performed based on the right gastroepiploic and right gastric arteries. Pyloroplasty, pyloromyotomy or no drainage was optional. Lymphadenectomies along the common hepatic artery, left gastric and splenic artery either *en bloc* or separately were performed and removal of sufficient crural fibres and a cuff of diaphragm performed if required for tumour clearance. The pericardial fat pad and strips of pleura were removed. Transection of the lesser curve could be undertaken during the abdominal or thoracic phase of the operation. Placement of a feeding jejunostomy or nasojejunal tube was at the surgeon’s discretion as was placement of intra-abdominal and intrathoracic drains. Procedures to minimize diaphragmatic herniation were permitted. The anastomotic technique and methods to close the incisions were at the surgeon’s discretion.

For both surgical approaches, the chest was opened through a right thoracotomy and the mediastinal pleura overlying the oesophagus was excised in continuity with the oesophagus. The posterior limit of the dissection was the antero-lateral wall of the aorta, so that the thoracic duct was mobilized with the oesophagus and perioesophageal tissues. The thoracic duct was tied on the aorta low in the chest cavity. The oesophagus was mobilized to the level of at least the aortic arch. Paraoesophageal nodes were removed in continuity with the oesophagus. Lymph nodes at the tracheal bifurcation, and along the right and left main bronchi to the pulmonary hilus, were removed *en bloc* or separately at the surgeon’s discretion. Excision of the paratracheal and recurrent laryngeal nodes was at the discretion of the surgeon and not mandated. The anastomotic technique was at the surgeon’s discretion.

### Outcomes

All items in the core outcome set for oesophageal cancer managed with surgery were measured^14^. The primary outcome was recovery of physical function, assessed using the validated patient-reported European Organization for Research and Treatment of Cancer quality of life questionnaire (EORTC QLQ-C30) at 3 and 6 weeks after surgery and 3 months after randomization^15^. The physical function scale is based on five questions, the score being transformed to a 0–100 scale with higher scores indicating better mobility and health. Longer follow-up on this and other patient-reported measures of health-related quality of life, costs and survival will be reported elsewhere.

The following secondary outcomes were recorded at days 3 and 6 after surgery for participants who were still in hospital and while the participants’ allocations were masked: forced expiratory volume in one second and forced vital capacity measured by spirometry, and patient-reported pain using a visual analogue scale^16^. The patient-reported EuroQoL EQ-5D-5L health-related quality of life questionnaire^17^ was collected at baseline, 6 days, 3 weeks, 6 weeks post-surgery and 3 months post-randomization. Survival and complications, reported using the standardized list of complications recommended by the Esophageal Complications Consensus Group^18^, were collected on a case report form for the 90 days following random allocation. The case report forms included the Consensus Group definitions of oesophagoenteric leak, conduit necrosis/failure, recurrent laryngeal nerve injury involvement and chyle leak severity; the Berlin definition of acute respiratory distress syndrome^19^; and the American College of Chest Physicians and Society of Critical Care Medicine Consensus Conference Committee definitions of generalized sepsis and multiple organ dysfunction syndrome^20^. In addition, pneumonia was defined as ‘New lung infiltrates plus clinical evidence that the infiltrate is of an infectious origin, which includes the new onset of fever, purulent sputum, leukocytosis or a decline in oxygenation’^21^. The impact of complications during the postoperative stay was assessed using the Clavien–Dindo scale^22^. The original plan to assess Clavien–Dindo at 30 days was amended to collect the data at discharge, to ensure all complications during the postoperative stay were included for all patients.

### Statistical analysis

Two hundred and three patients recruited to each of the hybrid and open surgery groups allowed a minimum clinically important difference of 0.4 s.d.^15,23^ on the primary outcome to be detected with >90% power at the two-sided 5% significance level, with up to 15% of patients not following their allocated procedure and 10% failing to complete the primary outcome.

The statistical analysis plan for the ROMIO study was written prior to the completion of study follow-up by members of the study team without access to the data^24^. The treatment effect on the primary outcome measure was estimated in an analysis following the intention-to-treat principle (comparing the two groups of patients as randomly allocated to hybrid or open surgery) as closely as possible. The treatment effect was quantified as the difference in mean score on the EORTC QLQ-C30 physical function scale across the three time points (with 95% c.i. and two-sided *P*), estimated as the coefficient of a binary variable distinguishing the two treatment groups, in a two-level linear regression model. The two levels accommodated the repeated measures design by separating variation between individuals from variation between each individual’s responses at the three outcome assessment points; this is a variance components model and assumes normal distributions for both the mean responses of individuals and for the individual responses on the outcome measure. Treatment centre, assessment point, whether the patient underwent neoadjuvant treatment and the baseline value of the outcome measure were also included as covariates. The two pilot-phase centres were each included as two separate centres in this analysis, distinguishing participants recruited in the pilot and main study phases. Physical function was assumed to be zero at an assessment point for any participants who had died or who were recorded as too ill to complete patient-reported outcomes (generally if they were known to be in an intensive care or high-dependency unit), but otherwise there was no imputation of missing data in the primary analysis and participants were included if at least one of the assessments was completed. Three subgroup analyses were pre-specified: whether a patient underwent neoadjuvant chemotherapy/chemoradiotherapy prior to surgery; POSSUM physiological risk score for post-surgery mortality and morbidity^25^ assessed at recruitment; BMI assessed at recruitment. Sensitivity analyses investigated the potential impact of any missing primary outcome data, investigated post-surgical pain among participants whose allocated surgery was completed (as participants undergoing a different surgical procedure are likely to be informed), and further adjusted the primary analysis for time between randomization and surgery.

Secondary outcome measures are presented as summary statistics according to the groups to which participants were randomly allocated. At the request of the editors the authors have added post-hoc hypothesis tests for post-surgical outcomes (chi-square and Wilcoxon rank sum tests as appropriate) and the Clavien–Dindo classification of complications (ordered logistic regression). Stata Statistical Software version 17.0 was used for all analyses (StataCorp 2021, College Station, Texas, USA).

### Economic analysis

The methods used to measure resource use in the pilot phases of ROMIO are too different from those used in the definitive trial to be combined. Therefore, the cost-effectiveness analysis includes only 328 patients recruited after the internal pilot phase. The methods were pre-specified in a health economics analysis plan^26^. EQ-5D-5L responses were converted to utilities using the National Institute for Health and Care Excellence (NICE)-recommended UK tariff^17,27,28^. These were combined with survival data to calculate quality-adjusted life years (QALYs), adjusted for differences in baseline EQ-5D-5L utility scores^26,29^. National unit costs were used to value resource use where available. Unit costs are reported in pounds sterling and, where applicable, were inflated to 2019/20 values. Key results were also presented in Euros using the exchange rate on 1 January 2020 (£1.00 = 1.18 Euros). A site survey identified typical theatre staff requirements for both procedures and key differences in reusable equipment and consumables. This information was combined with data (for example, duration of surgery) collected on the case report form (CRF) to micro-cost surgery. The CRF also recorded details of ICU stay, ward stay and re-interventions during the initial hospitalization and readmissions up to 3 months. Use of primary and community care after discharge was collected from patients using a resource-use questionnaire at 3 months^26^.

The cost-effectiveness analysis at 3 months was performed on an intention-to-treat basis from an NHS perspective. We used multilevel linear regression models, clustered by treatment centre, and with neoadjuvant treatment and the baseline EQ-5D-5L score as covariates to estimate the incremental cost, QALYs and Net Monetary Benefit (iNMB) of hybrid *versus* open oesophagectomy. The iNMB was estimated based on the lower willingness-to-pay threshold (that is, £20 000 per QALY) used by NICE to determine cost-effectiveness^27^.

Multiple imputation was applied for missing cost and EQ-5D-5L data. The primary economic analysis includes all patients randomized in the main phase of the trial, including those where costs and/or outcomes have been estimated by imputation. The authors present a cost-effectiveness acceptability curve to depict the probability that hybrid surgery is more cost-effective than open oesophagectomy at various willingness-to-pay thresholds.

Sensitivity analyses estimated cost-effectiveness in cases with complete cost and outcome data and extended the perspective to NHS and social care costs.

## Results

### Patients

**Fig. 1:**
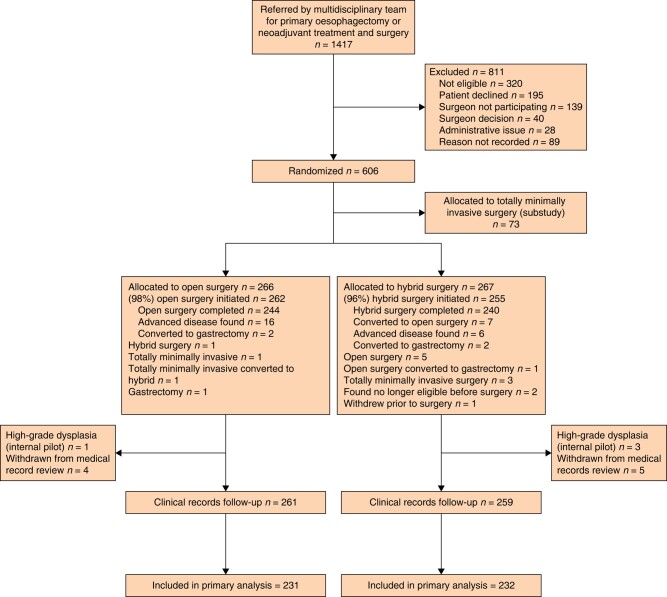
CONSORT flow chart

In total, 1417 patients were referred by their multidisciplinary team for oesophagectomy (*Fig. 1*), 811 of whom were not recruited to the study. The most common reasons recorded for this being not meeting the eligibility criteria (*n* = 320), the patient declining to take part (*n* = 195) and the patient’s surgeon not participating in ROMIO (*n* = 139). Details of ineligibility were available for 263 individuals from screening logs at one feasibility site plus all definitive study sites, where the most common reasons were not being suitable for all study procedures (*n* = 49), advanced stage (*n* = 42), previous procedures precluding a minimal access approach (*n* = 40), insufficiently fit for surgery (*n* = 39), extensive gastric involvement (*n* = 29), suitability not confirmed by the multidisciplinary team (*n* = 22) and co-morbidities (*n* = 14). Of the 1417 patients, 606 (43%) were randomly assigned, 266 to open, 267 to hybrid and 73 to totally minimally invasive oesophagectomy (to be reported separately). *Table S1* presents the numbers randomized to open or hybrid surgery by recruiting centre. Two patients allocated to open surgery withdrew all their data from the study and a further four patients (one allocated to open, three to hybrid surgery) had a diagnosis of high-grade dysplasia; these six patients are not included in the results presented here. At the completion of the internal pilot 199 participants had been recruited; due to these participants being recruited and treated at just two centres, and due to some measures, notably resource use, not being collected during the pilot, the authors agreed with the funder that they would exceed the target to ensure that at least 300 participants were recruited during the definitive phase.

The open and hybrid groups were well balanced in terms of sociodemographic and clinical characteristics at the time of allocation (*Table 1*). Most participants were recruited after completing neoadjuvant treatment. The two allocated groups were also comparable on disease characteristics (*Table 1*).

**Table 1:** Baseline patient and disease characteristics by allocated surgery

All randomized participants	Open surgery (*n* = 266)	Hybrid surgery (*n* = 267)
**Sex**		
Male	227	225
Female	39	42
Age (years), mean (s.d.)	66 (9)	67 (9)
BMI (kg/m^2^), mean (s.d.)	27 (4)	27 (4)
POSSUM physiological score, mean (s.d.), n*	18 (4) *n* = 187	18 (4) *n* = 189
Number WHO performance status score 1+, (%)*	65/200 (32)	64/196 (32)
**Neoadjuvant treatment; *n* (%)**		
Chemotherapy	176 (66)	170 (64)
Chemoradiotherapy	45 (17)	49 (18)
None	45 (17)	48 (18)
**Tumour histologic findings; *n* (%)†**		
Adenocarcinoma	237 (90)	235 (89)
Squamous cell carcinoma	25 (9)	26 (10)
Adenosquamous	2 (1)	2 (1)
High-grade dysplasia (internal pilot)	1	3 (1)
**Location of tumour in oesophagus; *n* (%)‡**		
Upper third	1	0
Middle third	8 (7)	25 (10)
Lower third/Siewert I	198 (75)	201 (77)
Junctional tumour, Siewert II	40 (15)	29 (11)
Junctional tumour, Siewert III	8 (3)	7 (3)
**Pretreatment clinical tumour category; *n* (%)†**,**‡**		
cT1	23 (9)	20 (8)
cT2	55 (21)	58 (22)
cT3	179 (67)	175 (66)
cT4a	8 (3)	11 (4)
**Pretreatment clinical nodal category; *n* (%)**		
cN0	128 (48)	109 (40)
cN1	98 (37)	119 (45)
cN2/cN3	40 (15)	39 (15)

*Not collected for pilot-phase participants at Plymouth. †Not recorded for one patient in each group as patient withdrew from routine data follow-up before collection. ‡Not recorded for two patients allocated to hybrid surgery due to withdrawal from routine data follow-up before collection.

### Surgery

Median time from allocation to surgery was 5 days (i.q.r. 2–12 days) in the open surgery group and 5 days (i.q.r. 1–10 days) in the hybrid surgery group. Allocated surgery was initiated for 262/266 (98%) of those in the open surgery group and 255/267 (95%) of those in the hybrid surgery group (*Fig. 1*). This included 16 patients allocated to open surgery and eight patients allocated to hybrid surgery who were found to have advanced disease preventing resection either before surgery was initiated (*n* = 2) or once surgery was underway (*n* = 22). For seven patients their allocated hybrid surgery was initiated but was converted to an open approach during surgery. Three patients in each of the allocated groups had a gastrectomy due to the tumour being found to extensively involve the stomach. Two patients, one allocated to each approach, were recorded as having a three-stage procedure (these data were not collected at one centre during the pilot phase). A feeding jejunostomy was inserted for 120 (45%) open surgery group and 111 (42%) hybrid surgery group participants, with this being done laparoscopically for 51 (46%) of the latter.

### Pathology

The median number of lymph nodes retrieved was 24 for patients in the hybrid group and 26 in the open surgery group, with 19 or more lymph nodes being retrieved in 75% of patients in both groups (*Table 2*). A greater proportion of participants with positive margins was observed in the open surgery group (31%) compared to the hybrid surgery group (24%), with R1 defined as ‘tumours equal to or less than 1 mm from the margin’ in accordance with the UK Royal College of Pathologists criteria^30^. However, this difference was consistent with chance. Other pathological staging parameters of the tumour were equally distributed following the two surgical approaches.

**Table 2:** Peri- and post-surgical measures

	Open surgery	Hybrid surgery	*P*
**Disease characteristics established post-surgery**			
Median lymph nodes with tumour* (Q1, Q3)†, *n*	0 (0–3)	0 (0–3)	
pStage, *n* (%)			
0	23 (10)	29 (12)	
I or II	106 (44)	109 (44)	
III	80 (33)	82 (33)	
IV	33 (14)	28 (11)	
**Measures of surgical performance**			
Median total lymph nodes retrieved* (Q1, Q3)†, *n*	26 (19–33)	24 (19–32)	0.449
Resection margin, *n* (%)			
R0	169 (69)	191 (76)	
R1	72 (30)	60 (24)	
R2	3 (1)	0	0.069
**Post-surgical outcomes**	***n* = 261**	***n* = 259**	
Median total hospital stay in days‡ (Q1, Q3)†	10 (8, 15)	11 (9, 16)	0.085
Median post-surgical hospital stay in days‡ (Q1, Q3)†	10 (8, 15)	11 (9, 15)	0.080
Mortality within 30-days post-surgery, *n* (%)	6 (2.3)	4 (1.5)	0.531
Mortality within 90-days post-surgery, *n* (%)	14 (5.4)	8 (3.1)	0.197
Death during initial hospital stay‡, *n* (%)*	4 (1.5)	4 (1.6)	0.987

*The two measures available for 244 and 252 participants allocated to open surgery and hybrid surgery, respectively. †Q1 and Q3 are the lower and upper limits of the interquartile range. ‡Not available for one participant allocated to hybrid surgery as not admitted to hospital.

### Secondary outcomes

Lung function and pain were comparable between the two allocated groups during the 6 days post-surgery (*Table S2*), with a per-protocol sensitivity analysis of the pain scores giving near-identical findings. A greater proportion of participants allocated to open surgery received pain relief at 3 days post-surgery from an epidural infusion, compared to the hybrid surgery group. Hospital stays were very similar between the two surgical approaches (median 10 *versus* 11 days), with 75% of participants in both allocated groups being discharged within 15 days of surgery (*Table 2*). Mortality due to any cause within 90 days of surgery was low and comparable to national audit data in each group.

The impact of complications during recovery, as assessed by the Clavien–Dindo classification, was comparable between the two allocated groups (*Table 3*), with about one-third of participants experiencing no complications, one-third complications of moderate severity requiring pharmaceutical intervention or similar and one-third more serious complications requiring an invasive intervention (Clavien–Dindo III or above). The occurrence of five key complications was comparable between the allocated groups with pulmonary infections being the most common, observed in about a third of participants. The need for an unplanned return to the intensive treatment or high-dependency unit or for further intervention during the post-surgical hospital stay was also closely comparable between the allocated groups. *Table S3* shows comparable outcomes for the full list of pre-specified complications during the 90 days following surgery. Deaths and intensive care resulted in the imputation of zero physical function scores at the 3-week (open surgery 9, hybrid surgery 8), 6-week (open surgery 8, hybrid surgery 4) and 3-month (open surgery 12, hybrid surgery 6) assessments of the primary outcome measure.

**Table 3:** Overall complication severity grading and counts of key complications and further interventions

All randomized participants continuing in follow-up	Open surgery (*n* = 261)	Hybrid surgery (*n* = 258)
**Clavien–Dindo classification of complications**		
Normal recovery, no complications	85 (33)	84 (33)
Grade I (minor deviation from normal course)	26 (10)	25 (10)
Grade II (pharmaceutical intervention)	62 (24)	67 (26)
Grade IIIa/IIIb (invasive intervention)	56 (21)	55 (21)
Grade IVa/IVb (life-threatening)	28 (11)	23 (9)
Grade V (death of patient)	4 (2)	4 (2)
* P for trend (ordered logistic regression)*	*0.783*	
**Key postoperative complications (any severity) within 30 days of surgery**		
Oesophagoenteric leak from anastomosis, staple line, or localized conduit necrosis	21 (8)	21 (8)
Conduit necrosis/failure	7 (3)	1
Chyle leak	10 (4)	11 (4)
Pneumonia/chest infection	91 (35)	85 (33)
Bleeding requiring intervention or transfusion	6 (2)	2 (1)
**Further intervention**		
Unexpected return to ITU/HDU during index hospitalization	35 (13)	30 (12)
Re-intervention requiring general anaesthetic during index hospitalization	42 (16)	49 (19)
Re-intervention requiring general anaesthetic within 90 days of surgery	43 (16)	49 (19)

Values are *n* (%). Counts are of patients who may have experienced more than one complication/intervention of a given type.

### Primary outcome

There was no evidence of a difference between hybrid and open surgery in recovery over the first 3 months post-random allocation, with the 95% c.i. excluding a clinically important difference in physical function (EORTC QLQ-C30 physical function scale, primary outcome) between the allocated groups (*Table 4*, difference in means = 2.3, 95% c.i. −1.7 to 6.4). Participants in both groups reported a negative impact on physical function following surgery, which despite some recovery of function was still apparent at 3 months.

**Table 4:** Physical function (EORTC QLQ-C30 subscale*) in the 3 months post-random allocation

	Open surgery (*n* = 261)		Hybrid surgery (*n* = 259)	
	Mean (s.d.)	*n*	Mean (s.d.)	*n*
Baseline	89 (15)	247	86 (17)	241
3 weeks post-surgery†	51 (25)	174	51 (27)	173
6 weeks post-surgery	61 (27)	201	61 (26)	209
3 months post-randomization	68 (27)	205	69 (25)	208
Treatment effect (adjusted difference in means, *n* = 231 *versus* 232)	2.3 (95% c.i. −1.7, 6.4) *P* = 0.256			

*Scores are between 0 and 100, with higher scores indicating better function. A positive treatment effect indicates better mean function with hybrid surgery. †Assessment point at 3 weeks post-surgery introduced during the internal pilot.

This same pattern of results was apparent when the analysis of the primary outcome measure was stratified by the internal pilot and main trial phases (*Table S4*). Greater resources were available during the definitive trial phase for monitoring the return of primary outcome questionnaires and sending reminders as necessary, these measures resulting in only 11 participants in the definitive trial phase not having primary outcome data compared to 46 during the internal pilot. Four of the 11 participants stopped completing questionnaire measures after they were found to be unsuitable for oesophagectomy, either before or during surgery, and an additional participant stopped after experiencing significant postoperative complications. Repeating the analysis of the primary outcome with the addition of time between randomization and surgery (median 5 days for participants in both allocated groups) as a further covariate resulted in the same estimated treatment effect.

### Subgroup analyses

The results of three pre-specified subgroup analyses are presented in *Table S5*. There was no evidence that the treatment effect on recovery during the first 3 months post-surgery was affected by whether the participant underwent neoadjuvant treatment pre-surgery, or by whether the participants physical condition was relatively poor (lower POSSUM Physiology scores). There was strong evidence that the relative benefits of open and hybrid surgery for recovery in the first 3 months post-surgery differed according to BMI (*P* = 0.004). Participants with a lower BMI had a faster recovery following hybrid compared to open surgery. This benefit of hybrid surgery was not seen in participants with higher BMI, where the observed difference, while consistent with chance, was in the direction favouring open surgery.

### Economic analysis

The cost of the initial procedure was marginally higher in the hybrid surgery arm due to greater equipment and consumable costs and a longer mean procedure time (*Table 5*). Higher ward and re-intervention costs in the hybrid surgery arm were largely offset by lower ICU and readmissions costs. Primary and other ambulatory care costs after discharge were very similar between study groups. Total health service costs at 3 months were somewhat higher in the hybrid surgery arm (£16 712 *versus* £16 304, or 19 740 *versus* 19 258 Euros), but confidence intervals were wide and included zero, indicating no strong evidence of cost differences (adjusted mean difference from the multilevel regression £206; 95% c.i. −£3381 to £3794). Mean (s.d.) EQ-5D-5L scores across both study groups improved from 0.389 (0.275) at 6 days to 0.660 (0.246) at 3 months (*Fig. S1*). However, differences in QALYs between study groups were small (adjusted mean difference from the multilevel regression −0.005; 95% c.i. −0.016 to 0.006). The incremental net monetary benefit at a willingness-to-pay threshold of £20 000 of hybrid surgery was small and negative −£367 (95% c.i. −£4010 to £3276; *Table 5*), providing no evidence that it is more cost-effective than open surgery at 3 months across a range of willingness-to-pay thresholds (*Fig. S2*). The cost-effectiveness of hybrid surgery was worse in sensitivity analyses limiting the analysis to cases with complete cost and outcome data (*Table S6*). Other sensitivity analyses included social care costs, and assumed quality-of-life scores were zero when participants were in ICU supported the findings of the primary economic analysis.

**Table 5:** Incremental costs, QALYs and net benefit of hybrid *versus* open surgery

	Open surgery (*n* = 162)		Hybrid surgery (*n* = 162)	
	Units*	Mean (s.d.), *n*	Units*	Mean (s.d.), *n*
Procedure equipment/consumables		£514 (£424), 162		£626 (£232), 162
Procedure staff time cost†	353 min	£2972 (£717), 162	364 min	£3067 (£662), 162
ICU	5.04 days	£7148 (£15 342), 162	4.72 days	£6446 (£12 545), 162
Ward	9.91 days	£4103 (£3413), 162	11.93 days	£4938 (£4757), 162
Re-interventions	0.18 procedures	£286 (£1293), 162	0.42 procedures	£708 (£2046), 162
Readmissions‡	2.40 days	£1166 (£3184), 162	1.89 days	£929 (£2918), 162
Total inpatient costs		£16 189 (£17 252), 162		£16 715 (£16 376), 162
Primary and other ambulatory§ care		£212 (£306), 141		£253 (£419), 139
Total health service cost		£16 304 (£16 094), 141		£16 712 (£15 007), 139
**EQ-5D-5L score**				
Baseline		0.822 (0.156), 162		0.812 (0.169), 160
6 days		0.398 (0.276), 143		0.381 (0.275), 143
21 days		0.578 (0.206), 127		0.546 (0.221), 133
42 days		0.629 (0.227), 134		0.638 (0.203), 137
90 days		0.655 (0.250), 143		0.665 (0.243), 145
QALYs		0.157 (0.042), 100		0.150 (0.045), 109
iNMB#	−£367 (95% c.i.: −£4010 to £3276) *P* = 0.84			

*Where applicable. †Time includes procedure time only; cost includes monitoring and recovery suite costs, if applicable. ‡Including costs of readmission for outpatient procedures. §Including emergency department. #Multilevel model after multiple imputation (*n* = 324) of missing costs and EQ-5D-5L scores.

## Discussion

In this pragmatic RCT comparing minimally invasive *versus* open surgery to treat localized oesophageal cancer, there was no evidence of differences in perioperative measures such as positive resection margins, the occurrence and severity of complications including chest infection, and no difference in post-surgical recovery as measured by patient-reported physical function during 3 months post-randomization (primary outcome). The 95% c.i. for the treatment effect on patient-reported physical function excluded the minimum clinically important difference of 10.4 points^23^. There was evidence that the relative benefits of open and hybrid surgery for post-surgical recovery differed according to the patient’s BMI, with hybrid surgery associated with faster recovery in those patients with a lower BMI. The marginally higher procedure costs of hybrid surgery were not offset by lower subsequent inpatient or ambulatory care costs. Differences in QALYs between study groups were small and there was no evidence that hybrid surgery was more cost-effective than open surgery at 3 months.

ROMIO has not replicated the findings of minimally invasive oesophagectomy reducing the occurrence of major complications within 30 days of surgery as reported by the MIRO study^7^, or reducing the occurrence of pulmonary infections during the hospital stay following surgery as reported in the TIME study^6^. Comparing participant characteristics between the three studies, the ROMIO participants are older (average age: ROMIO 67 years, MIRO 61 years, TIME 62 years), slightly heavier (average BMI: ROMIO 27 kg/m^2^, MIRO 25 kg/m^2^, TIME 25 kg/m^2^) and with a higher prevalence of adenocarcinoma (ROMIO 89%, MIRO 59%, TIME 62%). In the MIRO study, a greater proportion of participants allocated to open surgery were disabled (WHO performance status score 1+: 49%) compared to those allocated to the hybrid surgery group (35%), which may have influenced the occurrence of and response to complications. In the ROMIO study, 32% of participants allocated to each of the allocated groups were classified as disabled by this measure. The risk of pulmonary complications in the open surgery groups of the TIME, MIRO and ROMIO studies were comparable, affecting approximately one in three patients. The reduction of pulmonary infection risk in the TIME study’s totally minimally invasive group to 12%, which was not seen in ROMIO’s hybrid surgery group, cannot entirely be explained by the chest phase also being conducted with minimally invasive methods in the TIME trial, as a smaller but still substantial risk reduction to 18% was seen in the MIRO study’s hybrid surgery group. The risk of major complications (Clavien–Dindo category II or above, primary outcome measure in the MIRO study) was slightly less in the ROMIO open surgery group (57%) compared to the same group in the MIRO study (64%), but again the reduction in risk of major complications in the MIRO study’s hybrid surgery group to 35% was not seen in the ROMIO study hybrid surgery group. Much of the reduction in major complications seen in the MIRO study was due to a lower risk of Clavien–Dindo category II complications with hybrid surgery (14%) compared to open surgery (36%); category II complications are those requiring a pharmacological intervention such as antibiotics. Notably, despite the substantial reduction in complications following hybrid surgery observed by the MIRO study, this was not reflected by patient-reported physical function at 30 days post-surgery (EORTC QLQ-C30 Physical Function, mean scores hybrid surgery 66 *versus* open surgery 64)^31^. Complications were assessed by the operating surgeons themselves in the MIRO study. Finally, it is acknowledged that the R1 rates are higher in both groups in the ROMIO study compared to the MIRO study, which reported an R1/2 rate of only 3.4%. This may be related to the different pathological definitions of R1, different frequencies of complete tumour regression between the studies, or that surgery was less radical in the ROMIO study compared to the MIRO study. If one or both of the latter two possibilities are the case, then survival differences between participants in the two trials would be expected, and this will be examined during analysis of the long-term data from the ROMIO study.

Strengths of the ROMIO study include its relatively large sample size, the pragmatic design and good participation (at least 55% of eligible patients agreed to participate), blinding of participants and care staff to allocation in the 6 days post-surgery and high retention rates. An examination of the impact of missing data was considered unnecessary, as 95% of participants recruited in the main phase of the trial were included in the primary outcome analysis, giving an estimated treatment effect very similar to the cohort as a whole (87% of all randomized participants completed the primary outcome). Allocated surgery was initiated for 97% of participants, completed for 91%, and high surgeon fidelity to the allocated approach—ensured by quality assurance of the surgery—provide a clear and unbiased comparison of the two surgical approaches. Consultation with patients identified the importance of a patient-reported measure of post-surgical recovery as the primary outcome, but also of conducting a study big enough to highlight any unexpected differences in survival. The economic analysis provides evidence for surgeons and policy makers on the relative costs and benefits of hybrid surgery.

Limitations include the participants being aware of the surgery they had undergone when completing the patient-reported primary outcome measure, meaning a placebo-type effect is possible. There is currently interest in new robot-assisted approaches to oesophagectomy^32^. However, these new approaches should only be utilized as part of an RCT until sufficient evidence of their effectiveness and safety has accumulated; until that point, the ROMIO results remain relevant to clinical practice.

The ROMIO pragmatic RCT did not confirm previous findings of a reduction in complications with minimally invasive approaches to oesophagectomy, and furthermore found no evidence of differences in short-term clinical outcomes or patient-reported recovery of physical function over 3 months between the hybrid and open approaches. The evidence of an advantage of hybrid surgery in patients with lower BMI requires further examination and replication before influencing clinical guidelines. It is not clear that the higher equipment and consumable costs of hybrid surgery are justified by better patient outcomes. These findings do not require surgeons who have a preference for one of the two approaches to change their practice.

## Collaborators

Khurshid Akhtar, Salford Royal NHS Foundation Trust. Bilal Alkhaffaf, Salford Royal NHS Foundation Trust. Arun Ariyarathenam, University Hospitals Plymouth NHS Trust. Kerry Avery, University of Bristol. Paul Barham, University Hospitals Bristol and Weston NHS Foundation Trust. Adrian Bateman, University Hospital Southampton NHS Foundation Trust. Chloe Beard, University of Bristol. Richard Berrisford, University Hospitals Plymouth NHS Trust. Jane M. Blazeby, University of Bristol. Natalie Blencowe, University of Bristol. Alex Boddy, University Hospitals of Leicester NHS Trust. David Bowrey, University Hospitals of Leicester NHS Trust. Tim Bracey, University Hospitals Plymouth NHS Trust. Rachel C. Brierley, University of Bristol. Kate Briton, Royal Infirmary of Edinburgh. James Byrne, University Hospital Southampton NHS Foundation Trust. James Catton, Nottingham University Hospitals NHS Trust. Ram Chaparala, Salford Royal NHS Foundation Trust. Sarah K. Clark, Royal Infirmary of Edinburgh. Tonia Clarke, Royal United Hospital Bath NHS Trust. Jill Cooke, University Hospitals of Leicester NHS Trust. Graeme Couper, Royal Infirmary of Edinburgh. Lucy Culliford, University of Bristol. Heidi Dawson, Royal Infirmary of Edinburgh. Chris Deans, Royal Infirmary of Edinburgh. Jenny L. Donovan, University of Bristol. Charlotte Ekblad, Royal United Hospital Bath NHS Trust. Jackie Elliott, Independent advisor, patient & Carer Perspective. David Exon, University Hospitals of Leicester NHS Trust. Stephen Falk, University Hospitals Bristol and Weston NHS Foundation Trust. Naheed Farooq, Salford Royal NHS Foundation Trust. Kirsty Garfield, University of Bristol. Daisy M. Gaunt, University of Bristol. Fran Gill, University Hospitals Bristol and Weston NHS Foundation Trust. Robert Goldin, Imperial College, London. Athanasia Gravani, University of Bristol. George Hanna, Imperial College, London. Stephen Hayes, Salford Royal NHS Foundation Trust. Rachael Heys, University of Bristol. Carolyn Hindmarsh, Salford Royal NHS Foundation Trust. Sandra Hollinghurst, University of Bristol. William Hollingworth, University of Bristol. Andrew Hollowood, University Hospitals Bristol and Weston NHS Foundation Trust. Rebecca Houlihan, University Hospitals Bristol and Weston NHS Foundation Trust. Benjamin Howes, University Hospitals Bristol and Weston NHS Foundation Trust. Lucy Howie, Royal United Hospital Bath NHS Trust. Lee Humphreys, University Hospitals Plymouth NHS Trust. David Hutton, University of Bristol. Rosina Jarvis, University Hospitals Bristol and Weston NHS Foundation Trust. Marcus Jepson, University of Bristol. Rebecca Kandiyali, University of Warwick. Surinder Kaur, University of Bristol. Philip Kaye, Nottingham University Hospitals NHS Trust. Jamie Kelly, University Hospital Southampton NHS Foundation Trust. Anni King, University of Bristol. Jana Kirwin, University of Bristol. Richard Krysztopik, Royal United Hospital Bath NHS Trust. Peter Lamb, Royal Infirmary of Edinburgh. Alistair Lang, Royal Infirmary of Edinburgh. Vivienne Lee, University Hospitals Bristol and Weston NHS Foundation Trust. Sally Maitland, Nottingham University Hospitals NHS Trust. Nicholas Mapstone, Salford Royal NHS Foundation Trust. Georgia Melia, Nottingham University Hospitals NHS Trust. Chris Metcalfe, University of Bristol. Rachel Melhado, Salford Royal NHS Foundation Trust. Aida Moure-Fernandez, University of Bristol. Beena Nair, Lancashire Teaching Hospitals NHS Foundation Trust. Joanna Nicklin, University Hospitals Bristol and Weston NHS Foundation Trust. Fergus Noble, University Hospital Southampton NHS Foundation Trust. Sian M Noble, University of Bristol. Abby O’Connell, University of Bristol. Stephen Palmer, University of Bristol. Simon Parsons, Nottingham University Hospitals NHS Trust. Kish Pursnani, Lancashire Teaching Hospitals NHS Foundation Trust. Nicola Rea, Royal Infirmary of Edinburgh. Fiona Reed, University Hospitals Plymouth NHS Trust. Caoimhe Rice, University of Bristol. Cathy Richards, University Hospitals of Leicester NHS Trust. Chris Rogers, University of Bristol. Grant Sanders, University Hospitals Plymouth NHS Trust. Vicki Save, Royal Infirmary of Edinburgh. Chas Shaw, University of Bristol. Michael Schiller, University Hospitals Bristol and Weston NHS Foundation Trust. Rachel Schranz, University Hospital Southampton NHS Foundation Trust. Vinutha Shetty, Lancashire Teaching Hospitals NHS Foundation Trust. Beverly Shirkey, University of Bristol. Jo Singleton, Royal Infirmary of Edinburgh. Richard Skipworth, Royal Infirmary of Edinburgh. Joanne Smith, University Hospitals Plymouth NHS Trust. Christopher Streets, University Hospitals Bristol and Weston NHS Foundation Trust. Dan Titcomb, University Hospitals Bristol and Weston NHS Foundation Trust. Paul Turner, Lancashire Teaching Hospitals NHS Foundation Trust. Sukhbir Ubhi, University Hospitals of Leicester NHS Trust. Tim Underwood, University Hospital Southampton NHS Foundation Trust. Cellins Vinod, Salford Royal NHS Foundation Trust. Ravinder Vohra, Nottingham University Hospitals NHS Trust. Elizabeth M. Ward, University of Bristol. Rhian Warman, Nottingham University Hospitals NHS Trust. Neil Welch, Nottingham University Hospitals NHS Trust. Tim Wheatley, University Hospitals Plymouth NHS Trust. Katie White, Royal United Hospital Bath NHS Trust. Robin A. Wickens, University of Bristol. Paul Wilkerson, University Hospitals Bristol and Weston NHS Foundation Trust. Alexandra Williams, Lancashire Teaching Hospitals NHS Foundation Trust. Rob Williams, University Hospitals of Leicester NHS Trust. Natasha Wilmshurst, University Hospitals Plymouth NHS Trust. Newton A.C.S. Wong, North Bristol NHS Trust.

## Key institutions

The University of Bristol (Methodology expertise and coordination, including from the Bristol Trials Centre). University Hospitals Bristol and Weston NHS Foundation Trust (Methodology expertise, coordination, sponsorship, participant facing activities). University Hospitals Plymouth NHS Trust (Methodology expertise, coordination, participant facing activities). Lancashire Teaching Hospitals NHS Foundation Trust, Nottingham University Hospitals NHS Trust, NHS Lothian, Royal United Hospital Bath NHS Trust, Salford Royal NHS Foundation Trust, University Hospitals of Leicester NHS Trust and University Hospital Southampton NHS Foundation Trust (Participant-facing activities).

## Supplementary Material

znae023_Supplementary_Data

## Data Availability

Following publication of the main results, anonymized individual patient data will be made available for secondary research through https://data.bris.ac.uk/data/, along with supporting documentation (the ROMIO study protocol and statistical analysis plan have been published elsewhere^9,24^, a data dictionary will be available). Access will be conditional on assurance from the secondary researcher that the proposed use of the data is compliant with the MRC Policy on Data Preservation and Sharing regarding scientific quality, ethical requirements and value for money.
