# Supplementary material for: Laparoscopic or open abdominal surgery with thoracotomy for patients with oesophageal cancer: ROMIO randomized clinical trial
Source: Br J Surg. 2024 Mar 25;111(3):znae023. doi: 10.1093/bjs/znae023 (PMC10961947; doi:10.1093/bjs/znae023)

**SUPPLEMENTARY TABLES AND FIGURES**

**Laparoscopic versus open abdominal surgery and thoracotomy for patients with oesophageal cancer: The ROMIO pragmatic parallel group randomised controlled trial**

Group authorship: **The ROMIO Study Group**

**Key institutions:**

The University of Bristol (Methodology expertise and co-ordination, including from the Bristol Trials Centre)

University Hospitals Bristol and Weston NHS Foundation Trust (Methodology expertise, co-ordination, sponsorship, participant facing activities)

University Hospitals Plymouth NHS Trust (Methodology expertise, co-ordination, participant facing activities)

Lancashire Teaching Hospitals NHS Foundation Trust, Nottingham University Hospitals NHS Trust, NHS Lothian, Royal United Hospital Bath NHS Trust, Salford Royal NHS Foundation Trust, University Hospitals of Leicester NHS Trust, University Hospital Southampton NHS Foundation Trust (Participant facing activities).

**Corresponding author:** Chris Metcalfe

Bristol Medical School: Population Health Sciences, University of Bristol, 1-5 Whiteladies Road, Bristol, UK, BS8 1NU

chris.metcalfe@bristol.ac.uk

**Supplementary Appendixes**

| **The ROMIO Study Group** | **3** |
| --- | --- |

**Supplementary Tables and Figures – Index**

| **Supplementary Table 1.** Number of patients randomised at each study recruitment centre | **5** |
| --- | --- |
| **Supplementary Table 2.** Post-surgical measures of lung function and pain. These data were collected from the participants recruited subsequent to the completion of the internal pilot phase | **6** |
| **Supplementary Table 3.** Treatment complications occurring within 90 days of surgery. Counts are of patients, who may have experienced one or more complication with any degree of severity | **7** |
| **Supplementary Table 4.** Physical function (EORTC QLQ-C30 subscale) in the three months post-random allocation, stratified by recruitment cohort. Scores are between 0 and 100, with higher scores indicating better function. A positive treatment effect indicates better mean function with hybrid surgery | **10** |
| **Supplementary Table 5.** Pre-defined sub-group analyses. | **11** |
| **Supplementary Table 6.** Economic sensitivity analyses | **12** |
| **Supplementary Figure 1**. EQ-5D-5L scores over 90 days follow-up, by allocation arm | **13** |
| **Supplementary Figure 2.** Cost-effectiveness acceptability curve | **13** |

**The ROMIO Study Group**

**Study Management Group membership**

*Chief investigators:* University of Bristol: Chris Metcalfe (2013 to 2016, and 2019 to study completion); University Hospitals Bristol and Weston NHS Foundation Trust: Paul Barham (2016 to 2019, now retired).

*Co-applicants:* University of Bristol: Kerry Avery, Jane Blazeby, Jenny Donovan, William Hollingworth, Chris Rogers; North Bristol NHS Trust: Dr Newton ACS Wong; University Hospitals Bristol and Weston NHS Foundation Trust: Dr Benjamin Howes; University Hospitals Plymouth NHS Trust: Richard Berrisford; Independent advisor, patient and carer perspective: Jackie Elliott.

*Other members of the Study Management Group:* University of Bristol: Natalie Blencowe, Rachel C Brierley, Lucy Culliford, Kirsty Garfield, Daisy M Gaunt, Marcus Jepson, Rebecca Kandiyali, Anni King, Elizabeth M Ward. University Hospitals Bristol and Weston NHS Foundation Trust: Rebecca Houlihan, Paul Wilkerson.

**Collaborators**

*Co-applicants for feasibility study only:* University of Bristol: Sian Noble; Imperial College: George Hanna, Robert Goldin; University Hospitals Bristol and Weston NHS Foundation Trust: Stephen Falk.

*Bristol Trials Centre & Bristol Surgical Trials Centre:* University of Bristol: Chloe Beard, Natalie Blencowe, Rachel C Brierley, Lucy Culliford, Kirsty Garfield, Aida Moure-Fernandez, Daisy M Gaunt, Rachael Heys, David Hutton, Marcus Jepson, Rebecca Kandiyali, Caoimhe Rice, Sandra Hollinghurst, Jana Kirwin, Beverly Shirkey, Anni King, Elizabeth M Ward, Robin A Wickens, Abby O’Connell, Athanasia Gravani, Surinder Kaur, Stephen Palmer, Chas Shaw.

*Study centres:* Lancashire Teaching Hospitals NHS Foundation Trust: Kish Pursnani (Principal Investigator), Vinutha Shetty, Paul Turner, Alexandra Williams, Beena Nair (Lead Pathologist); Nottingham University Hospitals NHS Trust: James Catton (Principal Investigator), Sally Maitland, Rhian Warman, Georgia Melia, Simon Parsons, Ravinder Vohra, Neil Welch, Philip Kaye (Lead Pathologist); Royal Infirmary of Edinburgh: Graeme Couper, Chris Deans, Peter Lamb (Principal Investigator), Nicola Rea, Sarah K. Clark, Kate Briton, Alistair Lang, Heidi Dawson, Jo Singleton, Richard Skipworth, Vicki Save (Lead Pathologist); Royal United Hospital Bath NHS Trust: Tonia Clarke, Charlotte Ekblad, Katie White, Lucy Howie, Richard Krysztopik (Principal Investigator) Newton Wong (Lead Pathologist); Salford Royal NHS Foundation Trust: Bilal Alkhaffaf (Principal Investigator), Khurshid Akhtar, Ram Chaparala, Naheed Farooq, Carolyn Hindmarsh, Cellins Vinod, Rachel Melhado (Previous Principal Investigator), Stephen Hayes (Salford Lead Pathologist), Nicholas Mapstone (Manchester lead Pathologist); University Hospitals Bristol and Weston NHS Foundation Trust: Andrew Hollowood, Rebecca Houlihan, Joanna Nicklin, Michael Schiller, Rosina Jarvis, Christopher Streets, Dan Titcomb, Paul Wilkerson (Principal Investigator), Natalie Blencowe, Jane Blazeby, Paul Barham (Previous Principal Investigator), Vivienne Lee, Fran Gill, Newton Wong (Lead Pathologist); University Hospitals of Leicester NHS Trust: Alex Boddy (Principal Investigator), David Bowrey, Jill Cooke, David Exon, Sukhbir Ubhi, Rob Williams, Cathy Richards (Lead Pathologist); University Hospitals Plymouth NHS Trust: Richard Berrisford (Principal Investigator), Arun Ariyarathenam, Lee Humphreys, Grant Sanders, Joanne Smith, Fiona Reed, Natasha Wilmshurst, Tim Wheatley, Tim Bracey (Lead Pathologist); University Hospital Southampton NHS Foundation Trust: James Byrne (Principal Investigator), Jamie Kelly, Fergus Noble, Rachel Schranz, Tim Underwood, Adrian Bateman (Lead Pathologist)

**Supplementary Table 1.** Number of patients randomised at each study recruitment centre

| **Recruitment centre** | **Number of patients randomised (total 533)** |
| --- | --- |
| University Hospitals Plymouth NHS Trust | 217 |
| University Hospitals Bristol and Weston NHS Foundation Trust & Royal United Hospitals Bath NHS Foundation Trust | 121 |
| Nottingham University Hospitals NHS Trust | 63 |
| University Hospitals of Leicester NHS Trust | 46 |
| University Hospital Southampton NHS Foundation Trust | 27 |
| Lancashire Teaching Hospitals NHS Foundation Trust (Preston) | 24 |
| NHS Lothian (Edinburgh) | 19 |
| Salford Royal NHS Foundation Trust | 16 |

**Supplementary Table 2.** Post-surgical measures of lung function and pain. These data were collected from the participants recruited subsequent to the completion of the internal pilot phase

|  | **Open surgery** | | **Hybrid surgery** | |
| --- | --- | --- | --- | --- |
| **FEV_1_, as a percentage of predicted**^1^ | **Mean (SD)** | **N** | **Mean (SD)** | **N** |
| Pre-randomisation | 93 (24) | 139 | 91 (21) | 130 |
| Three days post-surgery | 48 (17) | 68 | 45 (19) | 68 |
| Six days post-surgery | 56 (19) | 71 | 48 (18) | 81 |
|  |  |  |  |  |
| **FVC, as a percentage of predicted^1^** | **Mean (SD)** | **N** | **Mean (SD)** | **N** |
| Pre-randomisation | 92 (24) | 139 | 89 (23) | 130 |
| Three days post-surgery | 47 (22) | 68 | 45 (20) | 68 |
| Six days post-surgery | 54 (19) | 71 | 49 (21) | 81 |
|  | | |  |  |
| **Pain: 0 is no pain, 100 is worst possible pain.** | **Median (Q1, Q3)**^2^ | **N** | **Median (Q1, Q3)**^2^ | **N** |
| Pre-randomisation | 0 (0, 1) | 159 | 0 (0, 3) | 158 |
| Three days post-surgery | 24 (10, 40) | 142 | 20 (6, 40) | 143 |
| Six days post-surgery | 22 (10, 48) | 151 | 20 (10, 50) | 147 |
|  |  |  |  |  |
| **Epidural infusion in place** | **n (%)** | **N** | **n (%)** | **N** |
| Three days post-surgery | 86 (54) | 160 | 61 (38) | 159 |
| Six days post-surgery | 12 (8) | 144 | 8 (6) | 142 |
|  |  |  |  |  |

SD = Standard deviation

1. Prediction for each participant based upon their age, sex and height according to equations in Table 2 of: E Falaschetti et al. Prediction equations for normal and low lung function from the health survey for England. Eur Respir J 2004; 23: 456-463.

2. Q1 and Q3 are the lower and upper limits of the inter-quartile range.

**Supplementary Table 3.** Treatment complications occurring within 90 days of surgery. Counts are of patients, who may have experienced one or more complication with any degree of severity

|  | **Open surgery (n=261)** | | **Hybrid surgery (n=258)** | |
| --- | --- | --- | --- | --- |
| **Key Complications** | **n** | **(%)** | **n** | **(%)** |
| Oesophagoenteric leak from anastomosis, staple line, or localised conduit necrosis. Severity type as follows: | 21 | (8.0) | 22 | (8.5) |
| I : no therapy change or medical treatment or dietary modification  II: interventional but not surgical therapy  III: surgical therapy | 7  2  12 |  | 8  3  11 |  |
| Conduit necrosis/failure | 7 | (2.7) | 1 | (0.4) |
| Chyle leak | 10 | (3.8) | 11 | (4.3) |
| Pneumonia / chest infection | 97 | (37.2) | 86 | (33.3) |
| GI bleeding requiring intervention or transfusion | 6 | (2.3) | 4 | (1.6) |
| **Pulmonary complications** |  |  |  |  |
| Pleural effusion requiring additional drainage procedure | 25 | (9.6) | 21 | (8.1) |
| Pneumothorax requiring treatment | 11 | (4.2) | 9 | (3.5) |
| Atelectasis mucous plugging requiring bronchoscopy | 4 | (1.5) | 1 | (0.4) |
| Respiratory failure requiring intubation | 20 | (7.7) | 14 | (5.4) |
| Acute respiratory distress syndrome | 6 | (2.3) | 6 | (2.3) |
| Acute aspiration | 1 | (0.4) | 1 | (0.4) |
| Tracheobronchial injury | 0 | - | 1 | (0.4) |
| Chest tube maintenance for air leak >10 days post-surgery | 3 | (1.1) | 2 | (0.8) |
| **Cardiac complications** |  |  |  |  |
| Cardiac arrest requiring CPR | 2 | (0.8) | 1 | (0.4) |
| Dysrhythmia atrial requiring treatment | 42 | (16.1) | 46 | (17.8) |
| Dysrhythmia ventricular requiring treatment | 7 | (2.7) | 8 | (3.1) |
| Congestive heart failure requiring treatment | 3 | (1.2) | 3 | (1.2) |
| Pericarditis requiring treatment | 0 | - | 2 | (0.8) |
| Myocardial infarction (confirmed by specialist team) | 2 | (0.8) | 1 | (0.4) |

**Supplementary Table 3 continued**

|  | **Open surgery (n=261)** | | **Hybrid surgery (n=258)** | |
| --- | --- | --- | --- | --- |
| **Gastrointestinal complications** | **n** | **(%)** | **n** | **(%)** |
| Ileus (small bowel dysfunction preventing or delaying enteral feeding) | 1 | (0.4) | 3 | (1.2) |
| Small bowel obstruction | 2 | (0.8) | 1 | (0.4) |
| Feeding Jej-tube complication | 12 | (4.6) | 12 | (4.7) |
| Pyloromyotomy / pyloroplasty complication | 1 | (0.4) | 0 | - |
| Clostridium difficile infection | 0 | - | 1 | (0.4) |
| Delayed conduit emptying requiring intervention or delaying discharge or requiring maintenance of NG drainage>7 days post-operatively | 20 | (7.7) | 21 | (8.1) |
| Pancreatitis | 2 | (0.8) | 3 | (1.2) |
| Liver dysfunction | 2 | (0.8) | 0 | - |
| Anastomotic stricture requiring endoscopic intervention | 5 | (1.9) | 1 | (0.4) |
| **Neurological / psychiatric complications** |  |  |  |  |
| Other neurological injury | 2 | (0.8) | 2 | (0.8) |
| Acute delirium | 20 | (7.7) | 23 | (8.9) |
| Delirium tremens | 2 | (0.8) | 0 | - |
| Recurrent nerve injury, all types and severity | 0 | - | 0 | - |
| **Post-operative infections** |  |  |  |  |
| Wound infection requiring opening wound or antibiotics | 17 | (6.5) | 12 | (4.7) |
| Central IV-line infection requiring removal or antibiotics | 6 | (2.3) | 1 | (0.4) |
| Intrathoracic / intra-abdominal abscess | 4 | (1.5) | 3 | (1.2) |
| Generalised sepsis | 11 | (4.2) | 15 | (5.8) |
| Other infections requiring antibiotics | 23 | (8.8) | 22 | (8.5) |
| **Post-operative wound / diaphragm complications** |  |  |  |  |
| Thoracic wound infection requiring draining | 7 | (2.7) | 3 | (1.2) |
| Acute abdominal wall dehiscence / hernia | 2 | (0.8) | 2 | (0.8) |
| Acute diaphragmatic hernia | 0 | - | 7 | (2.7) |

**Supplementary Table 3 continued**

|  | **Open surgery (n=261)** | | **Hybrid surgery (n=258)** | |
| --- | --- | --- | --- | --- |
| **Urological complications** | **n** | **(%)** | **n** | **(%)** |
| Acute renal insufficiency (doubling of baseline creatinine) | 7 | (2.7) | 8 | (3.1) |
| Acute renal failure requiring dialysis | 4 | (1.5) | 3 | (1.2) |
| Urinary tract infection | 6 | (2.3) | 6 | (2.3) |
| Urinary retention requiring reinsertion of urinary catheter | 6 | (2.3) | 7 | (2.7) |
| **Thromboembolic complications** |  |  |  |  |
| Deep venous thrombosis | 1 | (0.4) | 3 | (1.2) |
| Pulmonary embolism | 6 | (2.3) | 4 | (1.6) |
| Stroke (CVA) | 0 | - | 0 | - |
| Peripheral thrombophlebitis | 1 | (0.4) | 0 | - |
| **Other post-operative complications** |  |  |  |  |
| Multiple organ dysfunction syndrome | 6 | (2.3) | 2 | (0.8) |
| Tracheostomy | 9 | (3.4) | 8 | (3.1) |
| Complications related to epidural (e.g. abscess or neurological problems) | 1 | (0.4) | 1 | (0.4) |

**Supplementary Table 4.** Physical function (EORTC QLQ-C30 subscale) in the three months post-random allocation, stratified by recruitment cohort. Scores are between 0 and 100, with higher scores indicating better function. A positive treatment effect indicates better mean function with hybrid surgery

|  | **Open surgery** | | **Hybrid surgery** | |
| --- | --- | --- | --- | --- |
| **Post-pilot study cohort** | **(n=162)** | | **(n=162)** | |
|  | Mean (SD) | n | Mean (SD) | n |
| Pre-randomisation | 89 (15) | 162 | 88 (15) | 160 |
| Three-week post-surgery | 51 (26) | 135 | 51 (26) | 139 |
| Six-week post-surgery | 62 (27) | 136 | 61 (26) | 141 |
| Three-month post-randomisation | 68 (28) | 145 | 68 (25) | 146 |
| Treatment effect: Adjusted difference in means (95% confidence interval) (n = 155 v. 158) | 1.5 (-3.4, 6.4) | | | |
| **Internal pilot study cohort** | **(n=99)** | | **(N=97)** | |
|  | Mean (SD) | n | Mean (SD) | n |
| Pre-randomisation | 87 (14) | 85 | 82 (20) | 81 |
| Three-week post-surgery | 50 (23) | 39 | 50 (29) | 34 |
| Six-week post-surgery | 58 (26) | 65 | 63 (26) | 68 |
| Three-month post-randomisation | 69 (24) | 60 | 71 (25) | 62 |
| Treatment effect: Adjusted difference in means (95% confidence interval) (n = 76 v. 74) | 4.9 (-2.1, 12.0) | | | |

SD = standard deviation

**Supplementary Table 5.** Pre-defined sub-group analyses on physical function.

|  | **Open surgery** | | **Hybrid surgery** | | Adjusted difference in means (95% CI)^1^ |
| --- | --- | --- | --- | --- | --- |
|  | Mean (SD) | n | Mean (SD) | n |  |
| **Neoadjuvant treatment** |  |  |  |  |  |
| 3 weeks post-surgery  6 weeks post surgery  3 months post-randomisation | 50 (26)  61 (27)  68 (28) | 146  167  175 | 51 (26)  62 (26)  68 (25) | 145  172  173 | 2.4 (-2.0, 6.8) |
| **No neoadjuvant treatment** |  | |  | |  |
| 3 weeks post-surgery  6 weeks post surgery  3 months post-randomisation | 54 (24)  59 (28)  70 (21) | 28  34  30 | 50 (31)  61 (28)  72 (25) | 28  37  35 | 2.1 (-7.7, 11.9) |
|  |  | | *Interaction p-value = 0.969* | | |
| **BMI >26kg/m^2^** |  | |  | |  |
| 3 weeks post-surgery  6 weeks post surgery  3 months post-randomisation | 56 (22)  66 (24)  72 (23) | 90  104  107 | 49 (27)  61 (27)  67 (27) | 98  116  111 | -4.4 (-9.8, 1.0) |
| **BMI 26kg/m^2^ or below** |  | |  | | |
| 3 weeks post-surgery  6 weeks post surgery  3 months post-randomisation | 46 (28)  55 (29)  64 (30) | 84  97  98 | 53 (26)  62 (24)  71 (23) | 75  93  97 | 10.1 (4.2, 16.0) |
|  |  | | *Interaction p-value = 0.004* | | |
| **POSSUM Physiology score > 17** | | |  | |  |
| 3 weeks post-surgery  6 weeks post surgery  3 months post-randomisation | 46 (26)  57 (26)  67 (26) | 92  112  112 | 50 (28)  60 (26)  67 (26) | 97  121  124 | 3.4 (-1.9, 8.8) |
| **POSSUM Physiology score 17 or below** | | |  | |  |
| 3 weeks post-surgery  6 weeks post surgery  3 months post-randomisation | 57 (24)  65 (27)  70 (28) | 82  89  93 | 53 (25)  64 (26)  72 (23) | 76  88  84 | 1.0 (-5.1, 7.2) |
|  |  | | *Interaction p-value = 0.864* | | |

SD = standard deviation; CI = confidence interval.

1. The difference between allocated groups on the primary outcome measure, the physical function scale of the EORTC QLQ-C30, with positive values favouring hybrid surgery.

**Supplementary Table 6.** Economic sensitivity analyses

|  | Open surgery (n=162) | | | Hybrid surgery (n=162) | | | Incremental net monetary benefit at £20,000 per QALY (95% CI), p-value |
| --- | --- | --- | --- | --- | --- | --- | --- |
|  | n | Mean | (SD) | n | Mean | (SD) |  |
| Sensitivity analysis 1: Complete case | | | | | | | |
| Health service cost | 141 | £16,304 | (£16,094) | 139 | £16,712 | (£15,007) | -£2,141  (-£4,202 to -£81)  p=0.04 |
| QALYs | 100 | 0.157 | (0.042) | 109 | 0.150 | (0.045) |  |
| Sensitivity analysis 2: Social care costs included | | | | | | | |
| Health and social service cost | 141 | £16,319 | (£16,094) | 139 | £16,730 | (£15,005) | -£364^1^  (-£4,006 to £3,279)  p=0.85 |
| QALYs | 100 | 0.157 | (0.042) | 109 | 0.150 | (0.045) |  |
| Sensitivity analysis 3: Quality of life assumed to equal zero for days in ICU | | | | | | | |
| Health service cost | 141 | £16,304 | (£16,094) | 139 | £16,712 | (£15,007) | -£372^1^  (-£4,033 to £3,289) p=0.84 |
| QALYs | 106 | 0.151 | (0.049) | 115 | 0.147 | (0.047) |  |
| 1 Multilevel model after multiple imputation (n=324) of missing costs & EQ-5D-5L scores | | | | | | | |

**Supplementary Figure 1**. EQ-5D-5L scores over 90 days follow-up, by allocation arm


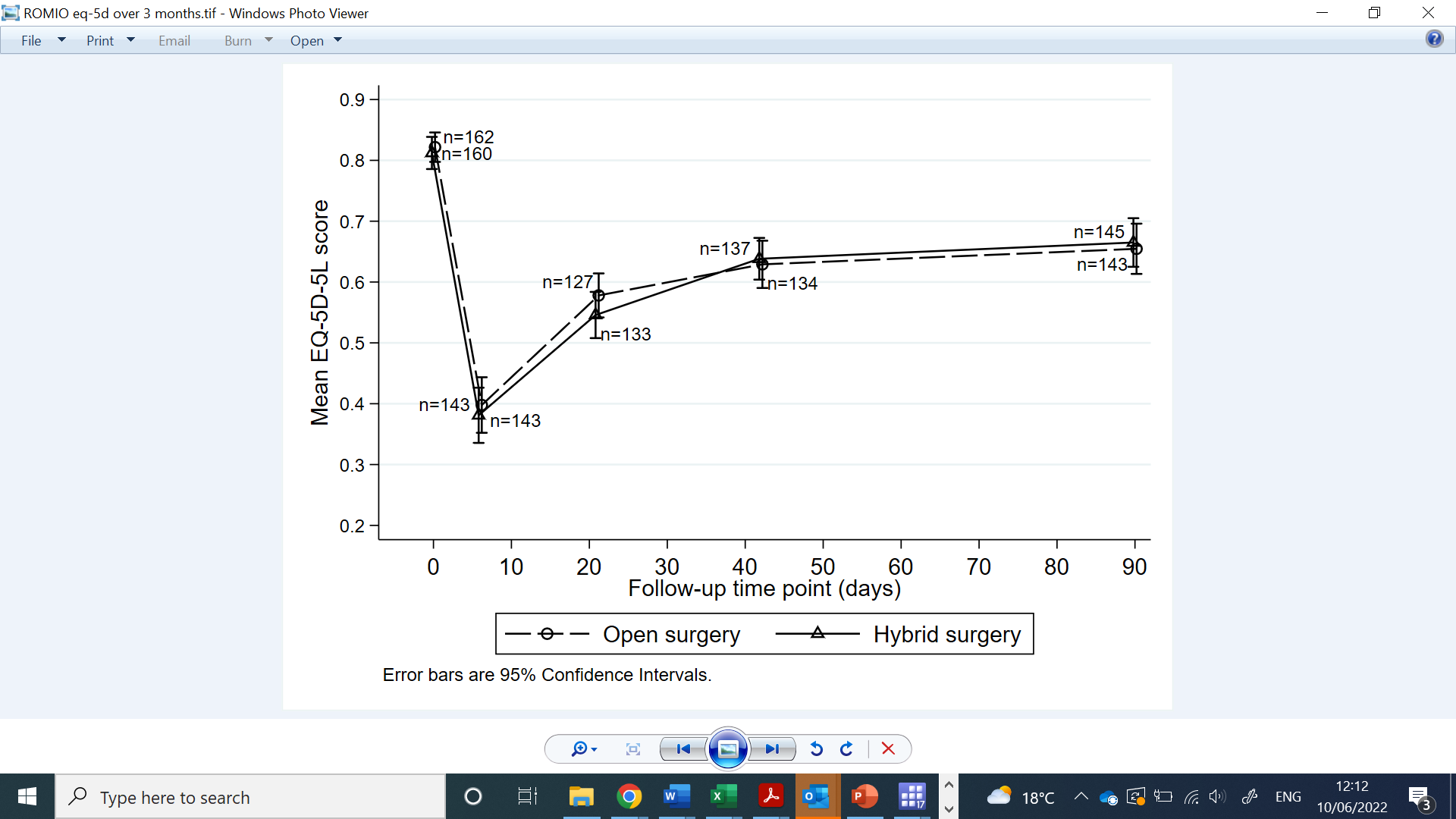


**Supplementary Figure 2.** Cost-effectiveness acceptability curve


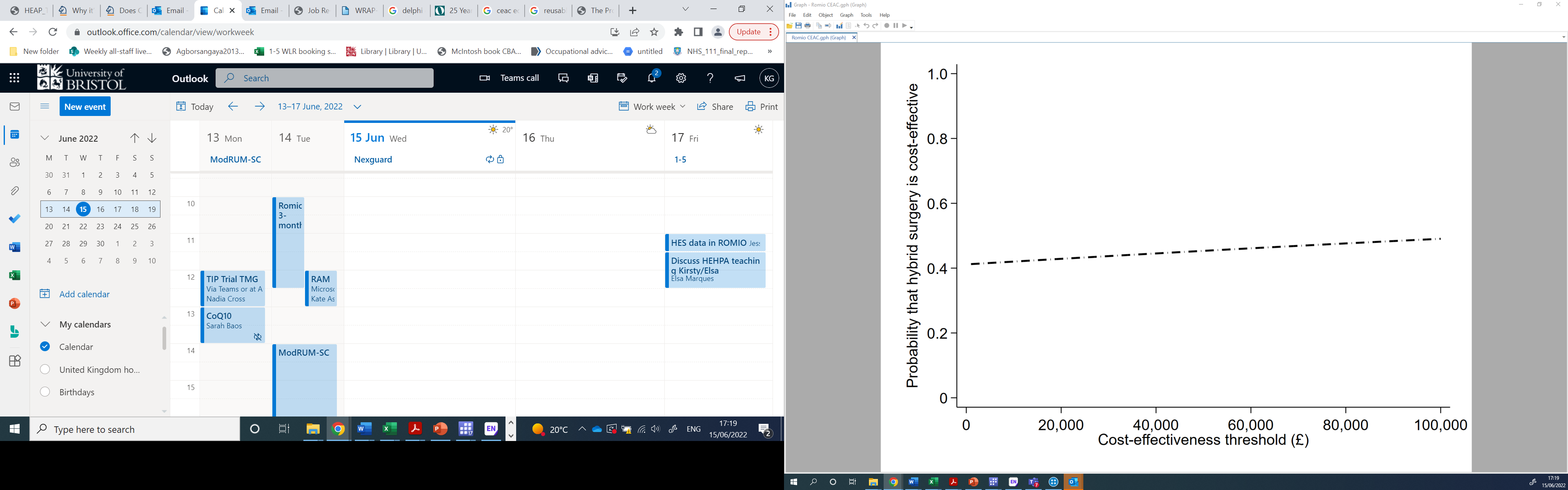

Supplement: znae023_Supplementary_Data [file znae023_supplementary_data.docx]
